# Integrating Digital Health Innovations to Achieve Universal Health Coverage: Promoting Health Outcomes and Quality Through Global Public Health Equity

**DOI:** 10.3390/healthcare13091060

**Published:** 2025-05-05

**Authors:** Mohamed Mustaf Ahmed, Olalekan John Okesanya, Noah Olabode Olaleke, Olaniyi Abideen Adigun, Uthman Okikiola Adebayo, Tolutope Adebimpe Oso, Gilbert Eshun, Don Eliseo Lucero-Prisno

**Affiliations:** 1 SIMAD Institute for Global Health, SIMAD University, Mogadishu 2526, Somalia; momustafahmed@simad.edu.so; 2 Faculty of Medicine and Heath Sciences, SIMAD University, Mogadishu 2526, Somalia; 3 Department of Public Health and Maritime Transport, University of Thessaly, 382 21 Volos, Greece; okesanyaolalekanjohn@gmail.com; 4 Department of Medical Laboratory Science, Neuropsychiatric Hospital, Aro, Abeokuta 110101, Ogun State, Nigeria; uthmanadebayo85@gmail.com (U.O.A.); bimpeadebayo2002@yahoo.com (T.A.O.); 5 Department of Medical Laboratory Science, Obafemi Awolowo University Teaching Hospital Complex, Ile-Ife 220282, Osun State, Nigeria; noaholaleke@gmail.com; 6 Department of Medical Laboratory Science, Nigerian Defence Academy, Kaduna 800001, Kaduna State, Nigeria; olaniyiadigun.oa@gmail.com; 7 The Royal (Dick) School of Veterinary Studies, University of Edinburgh, Edinburgh EH8 9YL, UK; gilberteshun8a@gmail.com; 8 Department of Global Health and Development, London School of Hygiene and Tropical Medicine, London WC1E 7HT, UK; don-eliseo.lucero-prisno@lshtm.ac.uk; 9 Center for Research and Development, Cebu Normal University, Cebu 6000, Philippines; 10 Center for University Research, University of Makati, Makati City 1644, Philippines

**Keywords:** digital health, universal health coverage, health equity, telemedicine, artificial intelligence, global health

## Abstract

Digital health innovations are reshaping global healthcare systems by enhancing access, efficiency, and quality of care. Technologies such as artificial intelligence, telemedicine, mobile health applications, and big data analytics have been widely applied to support disease surveillance, enable remote care, and improve clinical decision making. This review critically identifies persistent implementation challenges that hinder the equitable adoption of digital health solutions, such as the digital divide, limited infrastructure, and weak data governance, particularly in low- and middle-income countries (LMICs). It aims to propose strategic pathways for integrating digital innovations to strengthen universal health coverage (UHC) and bridge health disparities in the region. By analyzing the best global practices and emerging innovations, this study contributes to the ongoing dialogue on leveraging digital health for inclusive, scalable, and sustainable healthcare delivery in underserved regions.

## 1. Background

According to the World Health Organization (WHO), universal health coverage (UHC) is a condition in which all individuals and communities receive essential healthcare services, including preventive, curative, rehabilitative, and palliative care, without suffering financial hardship [1]. This ensures that people can access the healthcare they need without facing economic consequences, such as catastrophic expenditures. UHC is central to Sustainable Development Goal (SDG) 3.8, which aims to ensure equitable access to healthcare services and medicines worldwide [2].

Despite its widespread recognition, achieving UHC remains challenging. Many countries, particularly low- and middle-income countries (LMICs), struggle with weak healthcare infrastructure, workforce shortages, and financial constraints that hinder progress toward universal access [3]. In many regions, out-of-pocket healthcare payments remain high, making essential services unaffordable for marginalized populations. For example, in sub-Saharan Africa and parts of South Asia, millions of people lack access to even the most basic healthcare services because of geographic, economic, and systemic barriers [4,5]. Additionally, the growing burden of non-communicable diseases (NCDs), infectious diseases, and emerging pandemics further strains already overburdened health systems, making it even more challenging to achieve equitable healthcare access [6].

In recent years, digital health innovations have emerged as a transformative force in global healthcare, offering novel solutions to bridge gaps in healthcare delivery [7,8]. Digital health refers to the use of digital technologies and information and communication systems in medicine and other health professions to enhance healthcare access, delivery, and outcomes, manage illnesses and health risks, and promote wellness [9,10]. This broad field encompasses a range of technologies, including telemedicine, artificial intelligence (AI), mobile health (mHealth), big data analytics, electronic health records (EHRs), wearable devices, blockchain technology, and digital therapeutics. These tools have revolutionized healthcare by enhancing accessibility, improving efficiency, and fostering patient-centered care (Table 1) [11,12]. For instance, telemedicine has significantly improved healthcare access by enabling remote consultations, reducing the need for physical travel, and connecting patients with healthcare providers regardless of location. This is particularly beneficial in rural and underserved areas, where specialist care is often scarce [13,14]. Similarly, AI-driven diagnostic and decision support systems assist healthcare providers in making more accurate and timely clinical decisions. AI-powered algorithms can analyze vast amounts of medical data, including imaging scans and genetic information, to detect diseases early, personalize treatments, and improve outcomes, especially in resource-limited settings where trained specialists may not be readily available [15,16,17]. These technologies are increasingly applied in specialty areas such as musculoskeletal imaging, where AI is used to enhance diagnostic accuracy and standardization [18,19].

Moreover, mHealth applications empower individuals by providing tools for self-monitoring, medication adherence, and chronic disease management [12]. Wearable devices, such as smartwatches [20,21] and biosensors [22], enable real-time health monitoring, allowing for the early detection of abnormalities and reducing hospital admissions [23]. Big data analytics play a crucial role in tracking disease patterns, predicting outbreaks, and optimizing healthcare resource allocation [24]. Blockchain technology enhances data security, ensuring patient privacy and seamless interoperability among healthcare systems [25].

Despite the promise of digital health, global health inequities remain a critical challenge. Although these innovations have the potential to improve healthcare accessibility and quality, many underserved communities face technological, economic, and infrastructural barriers that limit their ability to leverage these advancements [26,27]. The digital divide, marked by disparities in Internet access, digital literacy, and affordability of digital tools, exacerbates existing health inequalities [28]. For example, in many LMICs, Internet penetration is still low, and access to smartphones and digital health tools remains out of reach for many populations [29]. Rural areas, in particular, face significant connectivity challenges, further limiting the impact of digital health interventions [30]. Additionally, variations in government policies, data privacy regulations, and interoperability issues pose major obstacles to the widespread adoption of digital health solutions in the country’s healthcare system. Many healthcare systems lack a unified digital infrastructure, leading to fragmented data collection and inefficient healthcare service delivery. Concerns regarding data security, ethical AI implementation, and equitable distribution of digital health resources must also be addressed to ensure that digital innovations contribute to health equity, rather than deepening existing disparities [31,32].

To achieve equitable UHC, it is essential to integrate digital health innovations inclusively, scalably, and sustainably into the health system. In this review, we present a comprehensive examination of digital health technologies across a range of clinical and public health domains to demonstrate their cumulative potential in advancing UHC. Our unique perspective emphasizes that without deliberate and equity-driven deployment strategies, digital health innovations risk reinforcing existing disparities, rather than alleviating them. Thus, this review not only highlights technological advancements but also critically examines systemic barriers and proposes solutions to ensure that digital health serves as a catalyst for global public-health equity. While these interventions vary in function and scope, ranging from AI-enabled diagnostics to blockchain-supported data governance, each serves as a strategic component of a broader and more integrated health ecosystem. By showcasing examples from rare diseases, communicable and non-communicable disease management, and mental health, we illustrate how digital innovations can collectively address the accessibility, affordability, and equity dimensions of UHC, particularly in low-resource settings.

## 2. Digital Health Applications in Rare Diseases

Rare diseases collectively affect over 300 million people worldwide, with more than 7000 identified conditions [33]. These diseases present unique challenges in diagnosis, treatment, and long-term management owing to their low prevalence and the consequent lack of medical awareness, research, and treatment options. Patients with rare diseases frequently experience delayed or incorrect diagnoses, difficulties in accessing specialized care, and a lack of effective therapies [34]. However, the emergence of digital health innovations is transforming the landscape of rare disease management by enhancing diagnostic capabilities, expanding access to specialized care, empowering patients, and accelerating research and drug development [35].

One of the most critical challenges in rare disease care is the extended diagnostic journey that many patients must undergo. It often takes years for individuals to receive an accurate diagnosis, leading to prolonged suffering and disease progression. This delay arises due to overlapping symptoms with more common conditions, limited awareness among healthcare providers, and the absence of widely available diagnostic tools. Digital health technologies address these issues by integrating AI and machine learning (ML) into diagnostic processes [36]. AI-driven tools analyze vast amounts of patient data, including genetic information and clinical symptoms, to identify patterns that may indicate rare diseases. Facial recognition software, such as Face2Gene, employs deep learning algorithms to detect distinctive facial features associated with certain genetic disorders, thereby significantly improving diagnostic accuracy. Additionally, AI-assisted genomic sequencing has revolutionized the identification of rare metabolic and genetic disorders, allowing earlier interventions and better patient outcomes [37]. Beyond diagnostics, digital health helps overcome geographical and economic barriers to receiving specialized care. Patients with rare diseases often face challenges in accessing expert healthcare, as specialists are typically concentrated in a few medical centers, often far from their place of residence [38]. Telemedicine platforms have emerged as powerful solutions, enabling remote consultations with experts, regardless of location. Through virtual appointments, patients can receive accurate assessments, treatment plans, and follow-up care without extensive travel [13]. This has been particularly beneficial for individuals in rural or underserved regions, who would otherwise have limited or no access to specialists in rare diseases. Moreover, wearable devices and remote monitoring technologies further enhance care by allowing the continuous tracking of symptoms and disease progression. These tools transmit real-time health data to medical professionals, enabling timely interventions and reducing the frequency of hospital visits [39].

Patient empowerment is another area in which digital health has had a significant impact. Rare diseases often come with immense emotional and psychological burdens as patients and their families navigate uncertainty, misinformation, and social isolation. Digital tools address these challenges by providing educational resources, community support networks, and self-management applications [40,41]. mHealth apps have been designed to help patients monitor their symptoms, track medication adherence, and access personalized health recommendations [7]. These platforms not only improve disease management but also enhance patient engagement in their care. Additionally, online communities and social networks, such as RareConnect, allow individuals affected by rare diseases to share their experiences, exchange knowledge, and offer emotional support. This sense of community is crucial for alleviating feelings of isolation and fostering resilience among patients and caregivers [42].

In parallel, digital health technologies are revolutionizing research and drug development for rare diseases. Traditional clinical trials face substantial hurdles, including limited patient populations, high costs, and lengthy approval processes [43]. However, digital innovations are accelerating these processes through advanced data analytics, AI-driven research, and blockchain-based medical records. AI has significantly improved drug discovery by identifying potential treatment targets through the analysis of genetic and protein structures [44,45]. Deep learning algorithms can process vast datasets to predict drug efficacy and identify promising compounds, thereby reducing the time and cost of developing new therapies. Platforms such as DeepMind’s AlphaFold have made groundbreaking contributions to the understanding of protein structures, which is essential for developing treatments for genetic disorders [46].

Moreover, digital health fosters greater participation in clinical research through decentralized trials and patient data sharing platforms [47]. Blockchain technology is being leveraged to ensure secure and transparent data exchange, enabling researchers to access real-world evidence while maintaining patient confidentiality [48]. Crowdsourced data collection through patient registries also plays a pivotal role in advancing research, allowing scientists to analyze disease patterns and identify novel therapeutic approaches. By integrating these technologies, researchers can accelerate drug development, improve clinical trial efficiency, and ultimately deliver life-saving treatments to patients more rapidly [49,50]. Despite these advancements, significant challenges remain in the widespread implementation of digital health solutions for rare diseases in clinical settings. One major obstacle is the digital divide, which exacerbates health disparities among the population [27]. Many underserved communities lack access to high-speed Internet, digital literacy, and the necessary infrastructure to leverage telemedicine and other digital tools [28]. Addressing these disparities requires targeted investments in digital infrastructure, education, and policies that promote equitable access to healthcare technology [51]. Furthermore, regulatory and ethical considerations must be carefully navigated to ensure responsible use of AI, protect patient data privacy, and maintain interoperability across digital health systems. Policymakers and healthcare stakeholders must work collaboratively to develop standardized regulations that facilitate the safe and effective use of digital health innovations while safeguarding patient rights [52,53].

As digital health continues to evolve, its potential to transform the care of patients with rare diseases is immense. By integrating AI-driven diagnostics, expanding telemedicine services, empowering patients through digital tools, and accelerating research through data-driven innovations, healthcare systems can bridge the critical gaps in rare disease management. However, realizing this potential requires a collective effort from governments, healthcare providers, technology developers, and patient advocacy groups to ensure that these innovations are accessible, inclusive, and sustainable in the long term. As these technologies advance, they have the power to not only improve the lives of those living with rare diseases but also to reshape the future of healthcare by making precision medicine a reality for all patients.

## 3. Digital Health Applications in Managing Communicable Diseases (CDs)

Recent advancements in real-time disease surveillance have leveraged AI, mobile data, and wearable technologies to enhance the early detection and monitoring of infectious diseases, particularly COVID-19. AI-driven predictive models analyze diverse datasets to detect early signs of disease emergence and predict potential outbreaks [54,55]. Wearable technologies and smartphone applications provide continuous monitoring of users’ health indicators, facilitating early symptom detection and prompt intervention [56,57]. A study of 3246 people demonstrated that smartwatch-based alerting systems could detect pre-symptomatic COVID-19 signals up to three days before symptom onset in 78% of cases (Table 2) [57]. Furthermore, smartphone applications for contact tracing have been useful in tracking people and their exposures to assist in stopping the transmission of the virus [58]. To ensure the responsible use of surveillance technologies, privacy and ethical considerations must be addressed [54,58].

Satellite imagery and Geographic Information Systems (GIS) are powerful tools for tracking and managing disease outbreaks. These technologies enable the analysis of environmental factors that influence vector–host interactions and pathogen activities [59]. Early outbreak detection and trend tracking are made easier by GIS-based syndromic surveillance systems’ ability to identify temporal and spatial abnormalities in illness patterns [60]. Cholera, Rift Valley Fever, and West Nile Virus are a few of the water- and vector-borne illnesses for which GIS has been effectively utilized [59]. This technology is a tremendous help to public health organizations in their efforts to control diseases because of its capacity to visualize and analyze epidemiological data.

Mobile-linked point-of-care (POC) diagnostic tools are increasingly becoming feasible solutions for resource-limited settings, particularly in sub-Saharan Africa. A systematic review and meta-analysis of these methodologies demonstrated intermediate diagnostic accuracy for various disorders, including malaria and schistosomiasis (Table 3) [61]. Although the aggregate pooled estimations showed room for improvement, these devices have significant potential in settings with limited laboratory infrastructure. Economic studies have shown that rapid diagnostic tests for HIV and malaria are cost-effective and provide additional benefits, such as reduced clinic visits and wait times [62]. One promising development in smartphone integration in point-of-care (POC) diagnostics is a smartphone-powered multiplex immunological test for syphilis and HIV [63,64]. These cutting-edge methods could change the future of POC diagnostics as smartphone usage rises worldwide, especially in high-disease-burden, low-income settings.

Virtual consultations have become a vital tool for treating infectious diseases, particularly during the COVID-19 pandemic. Electronic consultations, also known as e-consults, provide rapid access to infectious disease specialists while lowering exposure risks and costs [71]. Telemedicine has expanded dramatically by enabling remote treatment and bridging the divide between urban and rural healthcare [72]. The rapid adoption of digital technologies in healthcare has enabled diagnosis, treatment, monitoring, and follow-up during the pandemic [73]. A sizable proportion of patients in rheumatology can be treated with teleconsultations because most patients only require dose titration or the continuation of existing treatment(s) [74]. Patients have expressed a preference for virtual consultations throughout the pandemic and were highly satisfied with the quality of teleconsultation [74]. These results imply that virtual care and telemedicine can successfully supplement traditional healthcare delivery, particularly in managing communicable diseases.

Social media platforms have emerged as crucial tools for digital health campaigns, providing opportunities to increase vaccine adherence and reach larger audiences [75]. Successful campaigns typically include visually appealing information, such as infographics and short videos; engage in storytelling; foster community involvement; and strategically employ hashtags. However, some studies have shown mixed results regarding increasing adoption rates [76]. These platforms can improve reactivity to new health issues and help digitize medicine by quickly sharing public health information [77]. It has been demonstrated that social media marketing can effectively promote certain health behaviors, such as flu vaccination, among college employees and students [78]. However, advertising should consider ideas such as social influence and recognize the crucial success aspects of social media to optimize its impact and the health belief model [75].

mHealth applications have the potential to improve vaccination uptake and management. Numerous studies have examined the use of smartphone applications for tracking, education, and vaccination. Applications that record and recall immunizations are the most common form; these are often designed for parents as part of more comprehensive health management tools [79,80]. Many applications use provenance information to increase data reliability and credibility [81]. Despite mobile apps and appointment reminders boosting vaccination uptake in low- and middle-income countries, barriers to general adoption remain [80,82]. These include a lack of translation, country-specific customizations, and disjointed offerings. Despite their potential, vaccination apps remain underutilized, possibly because of these limitations [83]. To overcome vaccine hesitancy, national and international health agencies should develop centralized and multilingual vaccination applications [80].

The integration of digital health technology into national health systems holds considerable promise for improving epidemic control. Digital public health monitoring can leverage real-time data from participatory systems, mobile networks, and social media to enhance early warning and outbreak detection capabilities [84]. The real-time exchange of clinicopathological data, enabled by unified digital surveillance systems, facilitates improved disease outbreak management and service delivery [85]. Digital health technology was crucial in supporting public health policy during the COVID-19 pandemic, as demonstrated by Catalonia’s implementation of a digital health plan [86]. Key technologies, including big data, artificial intelligence, cloud computing, and 5G, have been beneficial in combating the pandemic, and China has been able to avoid a second wave thanks to the deep integration of digital technology and public health [87]. Data delays, fragmentation, and privacy issues remain challenges [87].

## 4. Digital Health Applications in Managing Non-Communicable Diseases (NCDs)

Wearable technology has become a potent instrument for encouraging healthy living and the early identification of health risks. These gadgets can track heart rate, sleep patterns, physical activity, and other health metrics [88]. They have demonstrated potential for blood pressure monitoring and the detection of cardiovascular conditions, such as atrial fibrillation [89,90]. Wearables enable individuals to manage their health by offering real-time feedback and individualized improvement methods [88]. Combining them with engagement tactics, such as financial or social incentives, increases their efficacy in encouraging physical activity and general well-being [89].

Additionally, by promoting self-management and disease prevention, these devices can help close health gaps and reduce medical mistrust among certain groups, such as African Americans [91]. As technology evolves, wearables are expected to transform healthcare delivery and improve patient outcomes. However, while these technologies enable preventive care and early intervention, they also raise concerns about overdiagnosis and overtreatment, especially when subtle variations are misinterpreted as pathological, potentially leading to unnecessary interventions or anxiety in patients [92].

Digital health interventions have demonstrated potential in encouraging behavioral changes to prevent chronic illnesses. Particularly in high-income environments, these interventions may be cost-effective [93]. To support dietary behavior modification, a variety of digital platforms have been used, including mobile applications, video games, Internet-based tools, and personal digital assistants [94]. Multiple behavior modification strategies, including goal setting, feedback, social support, and self-monitoring, are frequently incorporated into effective interventions (Table 4) [95]. Clinically meaningful weight loss has been most successfully achieved using digital features that assist with behavior tracking, online coaching, and health education [95]. Digital interventions should investigate the entire spectrum of device functioning and their use in everyday self-management to optimize their impact (Table 4) [96]. Future research should focus on personalizing interventions and integrating behavior change techniques to promote long-term adherence [94].

The use of Internet of Things (IoT)-enabled devices for the remote monitoring of NCDs has drawn considerable interest in medicine. These systems use mobile phones and wearable sensors to gather vital sign data, which are then sent to cloud platforms for analysis by medical professionals [102,103]. These technologies enable real-time monitoring of diseases such as diabetes and hypertension, thereby improving patient management and enabling prompt interventions [104]. Data security, privacy, and interoperability are among the issues that IoT-based health monitoring systems must address [103,104]. Ontology-driven systems and semantic models have been suggested as solutions to these problems to improve data interpretation and interoperability [103]. Remote monitoring systems have the potential to improve NCD prevention, diagnosis, and treatment, potentially leading to better patient outcomes and lower healthcare costs, despite certain obstacles [104,105].

Patients with chronic diseases may benefit from improved medication adherence and health outcomes through mobile health applications. According to studies, these applications can greatly improve the adherence of patients with hypertension to their medications [106]. Refill reminders, medication tracking, configurable reminders, and the ability to store health information are essential components of successful medication adherence apps [107,108]. Some sophisticated systems integrate personal health monitoring and medication compliance management, gathering vital sign data in real time and sending them to healthcare practitioners via wireless biosensors and mobile phones [109]. This integration enables remote monitoring and modification of medication regimens. Users value apps with configurable prescription schedules, support for in-person visits, and capacity to track multiple health metrics [107]. However, technical problems and rigidity in dosage scheduling remain for some applications [107].

ML and AI are transforming personalized medicine by providing customized treatment regimens based on individual health information. To produce precise diagnoses and maximize treatment plans, these technologies examine a large amount of patient data, including genetic predispositions, lifestyle factors, and medical history [110,111]. By recognizing patterns, forecasting the probability of a condition, and suggesting tailored treatment choices, machine learning algorithms can greatly enhance patient outcomes and diagnostic accuracy [111,112]. The basis for creating individualized treatment regimens is the integration of several data sources, including clinical history and genomics [112]. Through effective subject stratification and decision-making optimization, multifunctional machine learning platforms can assist physicians [113]. Although challenges exist, including data isolation and ethical implications, the implementation of AI in healthcare has the potential to significantly improve personalized and population-based medicine at a lower cost [110,113].

Digital therapeutics show promise in managing NCDs and mental health conditions. They can complement traditional care by facilitating data exchange, improving disease monitoring, and promoting patient empowerment [114]. For chronic diseases and cancer, health provider-directed digital interventions such as web-based consultations and Internet-based cognitive behavioral therapy have been effective [115]. During the COVID-19 pandemic, digital health interventions supported NCD management with the frequent implementation of telemedicine and targeted client interventions [116]. Computerized cognitive behavioral therapy has been effective for anxiety and depression in adolescents and young people, particularly when combined with in-person elements [117]. However, the effectiveness of other digital mental health interventions remains inconclusive, and their cost-effectiveness and applicability in low-resource settings require further investigation [117]. Integrating digital therapeutics into existing healthcare systems and addressing adoption barriers are crucial for maximizing their potential [114].

Digital platforms offer promising solutions for delivering mental healthcare, addressing resource limitations, and improving access. Teletherapy, virtual care platforms, mobile applications, and wearable devices have revolutionized service delivery, eliminating geographical barriers and providing convenient access to therapy [118]. Research indicates that digital health interventions, including synchronous and asynchronous communication, computerized therapy, and cognitive training, can be effective for various mental health conditions [119]. Internet-based cognitive behavioral therapy (iCBT) has shown effectiveness comparable to that of clinician-delivered CBT for a range of conditions [120]. However, challenges remain, including the digital divide, privacy concerns, and the need to regulate mental health apps [118]. Further research is needed to evaluate the efficacy and safety of digital interventions, particularly for understudied mental health conditions and low-income countries [119,120].

Virtual assistants and AI chatbots are becoming increasingly promising tools for promoting emotional and mental health. These AI-powered tools offer tailored treatments for a range of mental health issues, such as substance use disorders, anxiety, and depression, using machine learning and natural language processing [121,122]. Chatbots offer benefits such as cost-effectiveness, scalability, and accessibility [122]. In addition to offering evidence-based tools and coping mechanisms, they can mimic human discussions and respond empathetically [123,124]. Usability problems, privacy concerns, and the requirement for human oversight persist despite research suggesting potential advantages in enhancing mental and emotional well-being [121,122,124]. To improve the efficacy of AI-driven mental health solutions, future research should focus on conducting large-scale trials, optimizing human-AI integration, and resolving ethical issues [122,124].

## 5. Challenges in Leveraging Digital Health for UHC and Global Public Health

**Figure 1:**
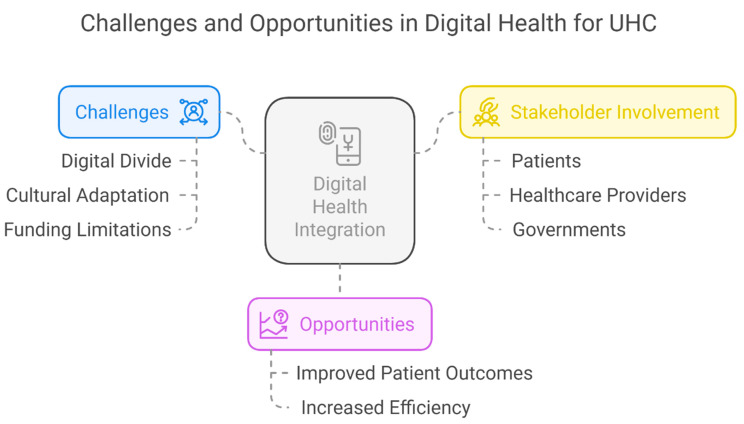
Challenges in leveraging digital health for UHC and global public health.

Over the years, efforts to achieve UHC in LMICs, particularly in Africa, have remained a top priority (Figure 1). This led to the African Union adopting the African Health Strategy (2016–2030) in 2016, which requires all African governments to ensure equitable healthcare for all citizens by 2030 [125]. This approach is in line with the 2030 Agenda for Sustainable Development, which stipulates that UHC guarantees that everyone with health needs has access to high-quality healthcare and that individuals receiving care do not experience financial hardship. The goal of UHC is to guarantee that everyone, particularly the most disadvantaged, has access to high-quality medical care without financial hardship. Financial risk protection and universal access to high-quality, safe, efficient, and reasonably priced basic healthcare services are among the goals of UHC [126,127].

Digital health, which integrates digital care programs and technologies into healthcare, daily living, and society, has been increasingly promoted as a means to enhance healthcare delivery, making medicine more precise and personalized, especially in the wake of the COVID-19 pandemic [128]. Again, as commonly referred to as digital healthcare, it makes use of digital information and communication technology to help people comprehend health issues and obstacles in more accurate and individualized ways while receiving medical treatment and social prescribing. mHealth apps EHRs, electronic medical records (EMRs), wearable technology, telehealth and telemedicine, and customized medicine are a few of the many types of technology that fall under this large umbrella term. The integration of hardware, software, networking, and sensors into healthcare delivery systems has promoted the digital revolution in the healthcare industry. This has transformed the industry and has numerous advantages for patients and caregivers [129].

The COVID-19 pandemic has disrupted health systems worldwide, and many LMICs have not been exempt. Before the COVID-19 pandemic, many of these nations had limited access to necessary health interventions [130]. The COVID-19 pandemic has demonstrated that the digital divide—the unequal distribution of access to digital technologies, including smartphones, tablets, computers, and the Internet—remains an unresolved public health issue. Due to the digital gap, many people were left out when many facets of life, including the provision of healthcare, switched to being done exclusively online in the early stages of the pandemic. In the United States, for instance, 20% of families experienced lockdowns without consistent access to broadband Internet connections. While many Latino and Black households relied on subscriptions that only worked on smartphones, others had no internet access. Internet connectivity is growing in some public places (such as shopping malls, schools, and parks), but its application in healthcare may be constrained by privacy concerns. Digital health usage is also influenced by how well each product fits into people’s daily lives and how well it meets their needs, situations, and worldviews. A lack of faith in technology, prejudice, and racism further exacerbates these disparities [131].

Health systems have begun to use digital health solutions to address this problem. Although startups in health technology have been established, they have had difficulty becoming mainstream and overcoming weak and unappealing healthcare systems in many LMICs. During the pandemic, digital health was used, and numerous nations with innovative digital infrastructures demonstrated that it could improve contact tracking, outbreak communication, lockdown reinforcement, logistical flow, and e-learning [130]. Despite significant progress in digital health information and communication technologies, several obstacles remain in fully mainstreaming digital health in LMICs, particularly during the COVID-19 pandemic. Prior to the crisis, many potential users of health technology in different regions of these countries were hesitant to adopt digital innovations in healthcare. This reluctance has made it difficult to enforce social distancing measures and other infection prevention and control (IPC) protocols across Africa [130]. A large portion of Africa and many other LMICs on other continents lack Internet connections, which makes it difficult to build digital infrastructure and discourages stakeholders from investing in digital health. The primary barriers to integrating and executing digital health efforts during the pandemic were pre-existing issues, such as a lack of funding incentives and priorities, problems with electrical supply and Internet connection, and a shortage of skilled personnel [130].

The European Regional Committee of the WHO recently adopted an action framework urging all Member States to seriously consider behavioral and cultural factors when designing health policies, including digital health interventions (DHIs). One effective approach to achieving cultural relevance is cultural adaptation, which involves systematically modifying an existing intervention to align with the cultural norms, beliefs, and values of the target population [131]. This approach is particularly critical when addressing the health inequities experienced by marginalized groups during digital health transitions [132]. Although funding has been allocated to address public health issues, the lack of health data in LMICs compared to developed countries poses another significant obstacle to utilizing digital health aimed at enhancing digital public health, making it impossible to plan, monitor, and evaluate interventions. Consequently, efforts to enhance universal health coverage have weakened [133]. Although digital health has many advantages, if it is not properly planned and executed, it may exacerbate existing health inequities. This is mostly because of differences in literacy, culture, and Internet connectivity. All parties involved in the matter must have faith in the digital services provided to foster trust in their use, as marginalized individuals and those subjected to prejudice may not trust digital healthcare technologies, including healthcare providers [134].

Some population groups have not fully adopted DHTs, despite significant investment and the growing number of DHTs available to healthcare consumers. These include members of lower socioeconomic classes and culturally and linguistically diverse (CaLD) communities, including immigrants, refugees, First Nations people, and racial or ethnic minorities. Research has consistently shown that DHT use and uptake are lower in people from CaLD groups than in non-CaLD individuals. For instance, research conducted in the United States has revealed that Latino and African American individuals are less likely than White Americans to use digital health technologies for medical purposes, while other studies have indicated that immigrants are less receptive to DHTs than non-immigrants. Due to access issues and the technology’s lack of cultural appropriateness, Indigenous and First Nations people are likewise less likely to use DHTs, which have the potential to exacerbate existing disparities in the healthcare system by causing a digital divide that compromises the efficient and fair provision of care. The inability to address the cultural, language, or health literacy requirements of these varied demographic groups may be one factor contributing to the poor uptake of DHTs. Understanding the viewpoints of end users (such as consumers, patients, and caregivers) regarding the variables impacting their use of digital health is crucial for developing DHTs [135]. Increasing the number of culturally appropriate digital health interventions (DHIs) is a strategic way to close these digital gaps [131].

## 6. Strategies for Bridging UHC and Global Public Health Equity Gaps Through Digital Health

**Figure 2:**
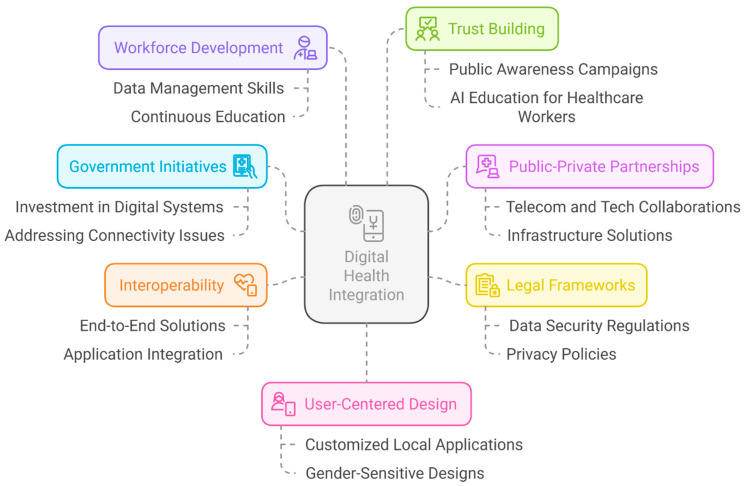
Bridging UHC and public health equity through digital health.

Investing more in evidence-based research, increasing clinical and field research, and gaining the support of various stakeholders are all necessary to envision and realize a seamless and healthier future through digital innovation (Figure 2). Given the revolutionary potential of digital health in achieving health goals, governments and their partners should prioritize it in UHC initiatives. This includes making increased investments in digitally enabled health systems a top priority, in addition to other important UHC investment areas. To ensure agreement with UHC goals, governments should create and execute digital health initiatives while incorporating partners and communities [136]. Additionally, these methods must address connectivity concerns, especially in rural and underserved areas, where health disparities are exacerbated by a lack of broadband access. Healthcare systems can be greatly impacted by private–public partnerships (PPPs) among governments, telecom companies, local technology enterprises, and financing institutions for the development and use of digital health products. Governments must concentrate on cultivating these collaborations and assisting local and commercial solutions that tackle problems such as infrastructure, human resources, economic viability, and the digital gender gap [137].

The establishment and implementation of legal frameworks are crucial for directing digital health (DH) systems in areas such as data ownership, availability, security, and consent. Governments must ensure that legislation, rules, and policies are sufficiently robust to enable successful and equitable digital health systems to be implemented. To enable people to use digital health technology responsibly, these policies should protect citizens’ rights, data privacy, and promote digital literacy [138]. Policies and procedures that safeguard people’s right to privacy and provide them with more control over how their data are used should be based primarily on these privacy concerns. Confidentiality, security, and data protection are frequently neglected in LMICs. Strong data security and privacy regulations must be established and implemented to protect patient records [139].

The development of end-to-end solutions and interoperability between applications is crucial, given the wide range of digital instruments used in healthcare.

## 7. Limitations

This paper is based on a narrative review of the literature and does not include systematic search or empirical data collection. As such, it may be subject to selection bias, and some relevant studies may have been unintentionally excluded from our analysis. The examples and technologies discussed were selected based on their prominence in the literature and relevance to UHC, but the scope is not exhaustive. Additionally, the rapid pace of digital health innovation means that new evidence and technologies may have emerged since this publication. The insights and recommendations provided should be interpreted within this context and may not be uniformly applicable to all settings, especially given the variability in digital infrastructure, policy environments, and healthcare capacity across countries.

Digital tools used for procurement, for example, should be able to interact with information about the larger supply chain at the health facility level. Increasing healthcare coverage and access for all populations can be achieved by improving the efficiency of health systems through the interoperability of digital tools [139]. The future workforce will require a wide range of talents that are currently unusual to support digitally enabled health in learning health systems. Clinicians, health system personnel, managers, and vendors require at least a basic understanding of data management, including data collection, storage, and normalization. In addition to basic proficiency in essential organizational systems, such as EHRs, interoperability, fundamental statistics, data science, data governance, teamwork, ethics, process improvement, and implementation science are also essential [140]. To maximize the advantages of digital technologies while reducing hazards, these digital treatments must incorporate ongoing monitoring, updates, and quality assurance procedures, in addition to the thorough training of PHC clinicians [141]. Public awareness campaigns and AI education for healthcare workers can significantly build trust and ensure effective utilization of these technologies. Essentially, what is at stake is the creation of a culture of trust that will enable all stakeholders in the big data ecosystem to benefit from the development of digital health technology. Trustworthy digital health activities require more than privacy protection. The key elements of trust include transparency, accountability, benefit sharing, and clarity regarding data ownership and control. Building trust is a multifaceted process, and achieving only one element is insufficient to build trust. A concerted effort is required to promote all these aspects. Consent innovation must be accompanied by mechanisms that clarify how individuals and communities will benefit from digital health developments, oversight systems that protect common interests, and accountability mechanisms to sustain public scrutiny [142]. Additionally, public policies must address the digital divide and consider citizens’ capacity to engage effectively with e-health initiatives [143].

In striving for better health outcomes, increased service quality, decreased costs, and better outcomes among both patients and providers, user-centered design is a critical element of any infrastructure strategy [144]. Local digital health applications are especially effective at removing barriers to healthcare access for vulnerable groups, such as rural residents and ethnic minorities, ensuring that even the most marginalized groups have access to high-quality healthcare services, which is crucial for closing healthcare equity gaps and advancing UHC [145]. For instance, these applications can be customized to address issues unique to these populations, such as language barriers, local healthcare needs, and access to mobile technology. The equal involvement of women and girls in digital health is impeded by several social and institutional barriers. Considering the social norms prevalent in the community, it is advantageous to actively involve women and girls in developing digital health intervention programs and delivery systems. For women and girls to effectively use digital health interventions, gender-sensitive designs must consider sociocultural contexts, literacy levels, and accessibility. In addition, nations should enact specific regulations to guarantee that vulnerable populations, such as those living in rural areas and ethnic minorities, have access to first-rate medical treatment.

## 8. Conclusions

To achieve equitable UHC, it is essential to integrate digital health innovations inclusively, scalably, and sustainably into health systems. This review examined the potential of digital technologies to address the longstanding gaps in accessibility, affordability, and quality of care, particularly in low- and middle-income countries. However, the successful implementation of this approach is not guaranteed. Persistent barriers, such as limited digital infrastructure, low digital literacy, and unequal Internet access, continue to hinder the meaningful adoption of telemedicine in many settings. Moreover, ethical challenges, data privacy concerns, and the risk of overdiagnosis from AI-based tools pose real threats if not properly addressed. Several digital health interventions have failed to scale because of a lack of contextual adaptation and stakeholder engagement. These examples underscore the need for participatory design, culturally tailored solutions, and transparent governance. By acknowledging both the opportunities and risks associated with digital health, this review provides a realistic roadmap for how these innovations can advance public health equity and support the realization of UHC.

## Figures and Tables

**Table 1:** Applications of digital health innovations in addressing challenges of rare diseases.

Digital Health Innovation	Application in Rare Diseases	Benefits	Examples
AI-Powered Tools	Early detection of genetic and metabolic disorders	Reduced diagnostic delays, improved accuracy	Face2Gene, DeepGestalt
Telemedicine Platforms	Remote consultations with specialists	Improved access to care, reduced travel burden	Rare Disease Clinical Research Network (RDCRN)
Wearable Devices	Continuous health monitoring and tracking biomarkers	Early identification of complications, real-time alerts	Smartwatches, fitness trackers with rare disease modules
mHealth Apps	Education, symptom tracking, and medication management	Enhanced patient self-management and adherence	MyRareTrack, RareGuru
Digital Patient Registries	Data collection for research and clinical trials	Accelerated therapy development, better patient outcomes	Global Rare Disease Patient Registry
Genomic Technologies	Identification of genetic mutations and therapeutic targets	Personalized medicine, precision drug development	CRISPR, whole genome sequencing
Crowdsourced Data Platforms	Patient-reported outcomes and real-world evidence	Enriched research data, faster drug discovery	PatientsLikeMe, RareShare
Virtual Reality (VR) Training	Simulated training for healthcare providers	Improved diagnostic and therapeutic skills	VR diagnostic modules for rare diseases
Blockchain in Health Records	Securing patient data and ensuring interoperability	Enhanced data privacy, seamless collaboration	MediLedger, HealthChain
Digital Biomarkers	Identification of digital patterns for early diagnosis	Non-invasive diagnosis and monitoring	Voice biomarkers for neurological rare diseases
3D Printing	Creation of personalized medical devices or prosthetics	Customized treatments, improved quality of life	3D-printed implants for skeletal dysplasias

**Table 2:** Wearable and mobile app-based early COVID-19 detection in 3246 participants [57].

Category	Details
Sample Size	3318 participants
Wearable Data Collected	2155 participants
Devices Used	Fitbit (1031), Apple Watch (970), Garmin watches (98), others (56)
COVID-19-Positive Cases	278 participants (84 confirmed via documentation (62 individuals) or verbal confirmation (22 individuals))
COVID-19 Positive with Wearable Data	84 participants (49 Fitbit, 35 Apple Watch)
Detection Performance	80% of COVID-19 cases identified at or before symptom onset
Asymptomatic COVID-19 Cases	18 participants, 8 had alert signals near test date
Real-Time Alerts	2117 participants received daily alerts
Pre-Symptomatic Detection	Median of 3 days before symptom onset
Diagnostic Methods	Real-time wearable data analysis with NightSignal, RHRAD, and CuSum algorithms
Key Symptoms Detected	Fatigue, aches and pain, headache, cough, fever
Vaccination Response	Alerts triggered post-vaccination; symptoms included fatigue, headache, and fever

**Table 3:** Diagnostic accuracy of mobile-linked point-of-care tools for disease detection in sub-Saharan Africa [61].

Disease	Sensitivity (95% CI)	Specificity (95% CI)	PPV (95% CI)	NPV (95% CI)	TP	FP	TN	FN	References
Schistosoma mansoni	50.0 (25.4–74.6)	99.5 (97.0–100)	85.7 (42.0–99.2)	97.3 (93.9–98.9)	51.0	0.5	0.51	50	[65]
Schistosoma haematobium	35.6 (25.9–46.4)	100 (96.6–100)	100 (86.7–100)	70.1 (63.1–76.3)	66.2	0.0	0.0	64.4	[65]
Schistosoma mansoni	68.2 (60.1–75.5)	64.3 (35.1–87.2)	95.4 (89.5–98.5)	15.8 (7.5–27.9)	32.2	35.7	36.2	31.8	[66]
Trichuris trichiura	30.8 (19.9–43.4)	71.0 (61.1–79.6)	40.8 (27.0–55.8)	61.2 (51.7–70.1)	71.5	29.0	29.0	69.2	[66]
Trichuris trichiura	54.4 (46.3–62.3)	63.4 (46.9–77.4)	85.1 (76.4–91.2)	26.5 (18.4–36.6)	46.4	36.6	37.2	45.6	[67]
Schistosoma haematobium	72.1 (56.1–84.2)	100.0 (75.9–100.0)	100.0 (86.3–100.0)	57.1 (37.4–75.0)	28.3	0.0	0.0	27.9	[68]
Malaria	80.2 (73.1–85.9)	100 (92.6–100.0)	100 (96.4–100.0)	65.6 (54.9–74.9)	20.0	0.0	0.0	19.8	[69]
Malaria	86.7 (79.3–92.2)	38.8 (33.6–44.1)	32.8 (27.7–38.3)	89.4 (83.4–93.8)	13.3	61.2	62.8	13.3	[70]

**Table 4:** Evidence from studies using video games and online education to promote dietary behavior change.

Summary of Results	Limitations	Strengths	References
Nutritional knowledge increased significantly; participants in the action stage of behavior showed superior effects; need for individualized games; shorter activities were preferred to ones with a longer commitment	Small exclusive groups already motivated to lose weight, self-report, and lack of follow-up and a control group	One of a few studies to investigate the effects of video games on BMI and nutritional knowledge	[97]
A decrease in fat mass and a shift toward a Mediterranean diet were observed post-intervention; the problematic effect of video games was not improved	Lack of control group, study limited to university students	Demonstrated the potential of video games in weight management	[98]
The experimental group reported a small increase in fruit and vegetable intake but the increase was not maintained at follow-up; there was no decrease in weight, but greater planning was observed in the intervention group	Self-selected attrition rates, self-reported eating measures and physical activity	Intervention content was individually tailored to increase adherence, satisfaction, and confidence in the intervention	[99]
Goal setting using online intervention increased intake of fruits and vegetables; goal setting was effective for behavior change but not for maintenance	Goal-setting functions were not assessed, options for goal-setting were limited, self-reporting and choice of a healthy population	One of the few studies where goal achievement was linked to dietary behavior change	[100]
Significant change to the Mediterranean diet; individual psychological preferences and readiness should be considered for an intervention	Lack of control group and randomization, self-reported dietary intake, self-selected participants, no attempt to compare cultural eating habits of different countries	First study to examine effects of online education on 4 social-cognitive constructs and study person-specific effects of interventions	[101]

## Data Availability

Not applicable because no new data or databases were used in the preparation of this work.
